# Noble classical and quantum approach to model the optical properties of metallic nanoparticles to enhance the sensitivity of optoplasmonic sensors

**DOI:** 10.1039/d2ra00824f

**Published:** 2022-06-07

**Authors:** Alemayehu Getahun Kumela, Abebe Belay Gemta, Tamirat Abebe Desta, Alemu Kebede

**Affiliations:** Adama Science and Technology University Adama Ethiopia alex.ph400@gmail.com

## Abstract

The bright light obtained from the quantum principle has a key role in the construction of optical sensors. Yet, theoretical and experimental work highlights the challenges of overcoming the high cost and low efficiency of such sensors. Therefore, we report a metallic nanoparticle-based metasurface plasmons polariton using quantum and classical models. We have investigated the material properties, absorption cross-section, scattering cross-section, and efficiency of the classical model. By quantizing light–matter interaction, the quantum features of light – degree of squeezing, correlation, and entanglement are quantified numerically and computationally. In addition, we note the penetration depth and propagation length from a hybrid model in order to enhance the optoplasmonic sensor performance for imaging, diagnosing, and early perception of cancer cells with label-free, direct, and real-time detection. Our study findings conclude that the frequency of incident light, size, shape, and type of nanoparticles has a significant impact on the optical properties of metallic nanoparticles and the nonlinear optical properties of metallic nanoparticles are dynamic, enhancing the sensitivity of the optoplasmonic sensor. Moreover, the resulting bright light shows the systematic potential for further medical image processing.

## Abstract 2

We report a metallic nanoparticle-based surface plasmonic polariton using quantum and classical models.

## Graphical abstract

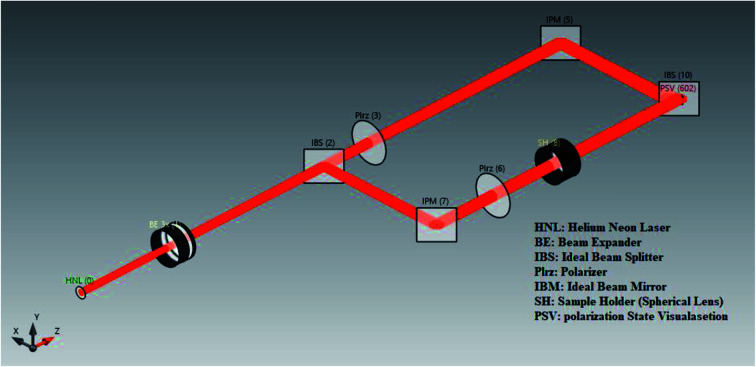

## 1 Introduction

Physicists consider the world we experience in terms of light and matter.^1,2^ This can be expressed by classical and quantum models for different applications. The classical model considers light as electromagnetic radiation and matter as a charged particle, in order to drive the propagation length and penetration depth of a surface plasmon polariton (SPP). While the quantum mechanical model describes phenomena by a quantization scheme^3–6^ for analytical and computational manipulations of quantum nature, such as quantum decoherence, quantum correlations (QCs), and quantum entanglement.^7–10^ The combinations of both models can help with the study of light–matter interactions for the enhancement of optical-based sensors.^11,12^

When light and metallic nanoparticles (MNPs) interact on the metal surface (MS), free electrons transit from the ground state to the excited state and *vice versa* (|g〉 ↔ |e〉) by an amount *ω*, depending on the intensity of incident light and meta-stable state of MNPs and MS. The collective oscillation of free electrons inside and outside of MS results from dipole oscillation. An oscillating dipole radiates fingerprint frequency since the particles have different numbers of electrons bound in different configurations.^13,14^ The resulting collective excitation of free electrons that propagate along a metal-dielectric interface is called a surface plasmonic polariton (SPP), which is 50% light and 50% particle, has a small mass, travels fast, and interacts strongly.^15–17^ The strong interaction makes both classical and quantum models best for expressing the optical properties of SPP.^18–20^

Linear and nonlinear interactions have been introduced for propagation length and interaction time enhancement.^21,22^ Using MNPs for difference frequency generation to induce extra noise and enhance propagation length.^23,24^ Enhancement of SPP properties has potential application in nanophotonics, security, super-resolution imaging, and optical biosensors.^25–27^ Plasmonic-based optical sensors (OPS) have high specificity, sensitivity, small size, and cost-effective advantages over conventional sensors. Their application has been used to measure cancer cell mechanics such as extracellular matrix, nucleoskeleton, and cytoskeleton; applying both the classical and quantum models of SPP properties.^28–31^

Recently, researchers have addressed different types of optical sensors for cancer treatment.^32–35^ Zakaria *et al.*^32^ have reported, a gas sensor to detect volatile organic compounds (VOCs) emitted from exhaled breath for early lung cancer diagnosis; their work provides high performance in distinguishing lung cancer from breast cancer with limited detection of other cancer cells. In the same year, Zhang *et al.*^33^ developed metamaterial biosensors in the terahertz frequency for theoretical and experimental investigation of apoptosis cancer cells, but developing metamaterials makes the work more difficult. Two years later Vural *et al.*^34^ proposed electrochemical biosensors using quantum dot (QD) nanocomposite materials for analyzing various cell types, but this requires the preparation of additional fluid. Adams *et al.*^35^ have reported electrical impedance spectroscopy to study the dynamics of cancer cells. All the above work encountered difficulties due to the complexity of the experimental work, cost-effectiveness, and specificity.^36–39^

Currently, many pioneers have focused on OPS for imaging, early diagnostics and post diagnostics of cancer cells, for its simplicity, high sensitivity, selectivity, and low cost: Bellassai *et al.* developed surface plasmon resonance (SPR) and localized SPR (LSPR)-based platforms for the detection of different classes of cancer biomarkers, but their work addresses only liquid-based samples and requires additional work to be cost-competitive, robust, and sensitive.^40^ One-year later Sojic *et al.* worked on enhancing the sensitivity of OPs using gold-coated micropillar-etched tips.^41^ However, all reported work on OPs is expensive, difficult to function, and less sensitive and selective for cancer diagnoses.

Therefore, this work aims to study the classical and quantum models of light–matter interactions to optimize OPS for high sensitivity using theoretical and computational expression. With MNPs arranged on a metal surface (Fe_2_O_3_) and ejecting intense coherent light, the results consider interactions by classically applying Maxwell equations and the modified Drude model; and quantum mechanically by quantization of the field, MNPs, and interactions. From the classical model the scattering cross-section, absorption cross-section extinction coefficient, and their efficiency are expressed. From the quantum mechanical model, we derive all the quantum properties of SPP by applying the Hamiltonian system. The combination of both models leads to the construction of a simple, cost-effective, highly sensitive, and selective OPS for cancer diagnosis.

## 2 Theories and parameters

### 2.1 Classical approach to model SPP properties

To model the properties of SPP classically, we applied Maxwell equations and Drude–Lorentz models. From the properties we determine, intensity, absorption cross-section, scattering cross-section, extinction cross-section and efficiency, with these parameters being tools to measure the capacity of a sensor.

The electric field resulting from light–matter interactions is given by the summation of the incident and output field. The output field is the difference between the incident field, absorbed, and transmitted field, defined by^42,43^

1 *E*(*ω*,*t*) = *E*_in_(*ω*,*t*) + *E*_out_(*ω*,*t*),

where the propagation of the incident laser field to the right and left (*E*_in_(*ω*,*t*) = *E*^+^_in_(*ω*,*t*) + *E*^¬^_in_(*ω*,*t*)) takes the form,2
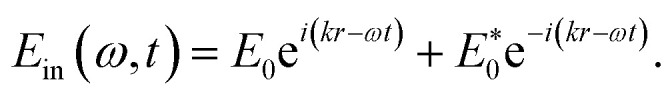
Here, *r* = *x̂* + *yĵ* + *zk̂* is the position in 3D, *ω* and *E*_0_ are the frequency and amplitude of the incident field, respectively, 
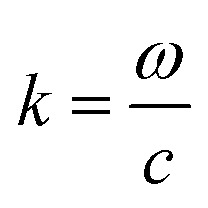
 is wave number and *c* is speed of light in a vacuum.

The output field (for details see Appendix A) takes the form,

3 *E*_out_(*ω*,*t*) = (*μ*_a_ + *μ*_b_)*E*_in_(*ω*,*t*).

where, 
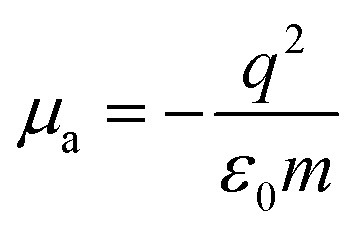
 and 
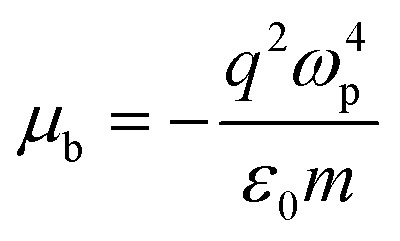
 are the transmission and absorbance dipole moment, respectively; plasma frequency, 
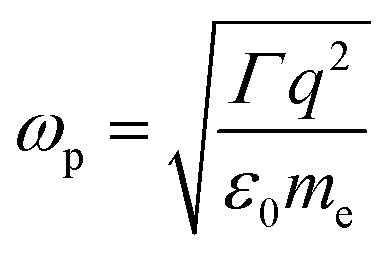
, is calculated using *ε*_0_ permittivity of free space, *m*_e_ mass of electron, *q* charge and 
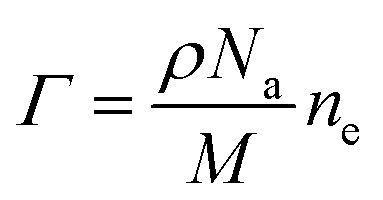
 ; in which, *ρ* is density, *N*_a_ is Avogadro’s number, *M* is the atomic mass of the particle and *n*_e_ is the number of free electrons.

Incorporating eqn (3) into eqn (1), the SPP field gives,

4 *E*(*ω*,*t*) = (1 + *ξ*)*E*_in_(*ω*,*t*).

Here, the decay rate of a polariton is a result of the incident coherent field

5 *ξ* = (*μ*_a_ + *μ*_b_).

#### 2.1.1 Linear and nonlinear response of light–matter interactions

Linear light–matter interactions occur when a single incident field produces a single photon as a result of a weak interactions. In other cases, if the matter is pumped by an intense incident field, more than a single photon is created *i.e.* a nonlinear response of light. Excitation of matter by either a weak or intense field results in the polarization of light, given by a summation of the linear and the nonlinear term^44^

6a *P*(*ω*,*t*) = *P*^L^(*ω*,*t*) + *P*^NL^(*ω*,*t*),

for

6b *P*^L^(*ω*,*t*) = *ε*_0_*χ*^(1)^*E*(*ω*,*t*),

and

6c *P*^NL^(*ω*,*t*) = *ε*_0_*χ*^(2)^*E*^2^(*ω*,*t*) + *ε*_0_*χ*^(3)^*E*^3^(*ω*,*t*) + … *ε*_0_*χ*^(*n*)^*E*^*n*^(*ω*,*t*)

where, first-order susceptibility (*χ*^(1)^) describes the linear optical properties including absorption and reflection. While *χ*^(2)^, *χ*^(3)^, and *χ*^(*n*)^ are second, third, and *n*^th^ order susceptibility, respectively, used for frequency conversion processes.

Thus, the collective movement of free electrons inside and outside of MS resulting from light-matter interactions is expressed by the modified Drude model in terms of polarization as reported by,^45^

7 −*ε*_0_*ω*_p_^2^*E*(*ω*,*t*) = *P̈*(*ω*,*t*) + *γ*_ab_*Ṗ*(*ω*,*t*) + *ω*_0_^2^*P*(*ω*,*t*)

where the left hand term is the driving force, *P̈*(*ω*,*t*) is the second derivative polarization (acceleration of plasma), *Ṗ*(*ω*,*t*) is the first order derivation of polarization (damping factor). While, *γ*_ab_ and *ω*_0_ are the damping coefficient and characteristic frequency, respectively.

Substituting eqn (4) into eqn (7) and applying differentiation with respect to time we find

8 *ε*_0_*ω*_p_^2^*E*(*ω*,*t*) = (*ω*^2^ − *ω*_0_^2^ + *iγ*_ab_*ω*)*ε*_0_*χ*^(1)^(1 + *ξ*)*E*(*ω*,*t*).

Applying simple rearrangement, the first order susceptibility takes the form9
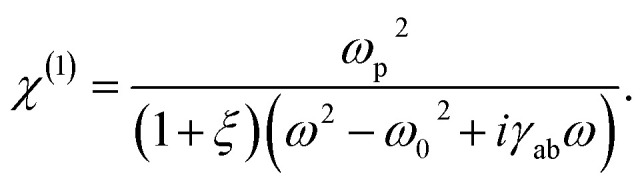


In addition, *χ*^(2)^ = 0 for crystals with a center of symmetry and optically isotropic optical materials, and *χ*^(3)^ = *β*(*χ*^(1)^)^4^ as defined in ref. 46 so third order susceptibility can be defined as10
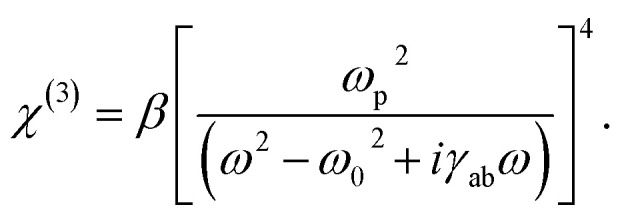
where *β* = 1 × 10^−7^ is the mean value constant.

#### 2.1.2 Dielectric function of materials

The dielectric function of materials is derived using Maxwell’s relation which connects the electric displacement field *D* with material properties as

11 *D*(*ω*,*t*) = *ε*_0_*E*(*ω*,*t*) + *P*(*ω*,*t*).

From this relation we can establish

12 *ε* = 1 + *χ*,

Using linear (eqn (9)) and the nonlinear susceptibility (eqn (10)) relation, the linear dielectric function is given by13
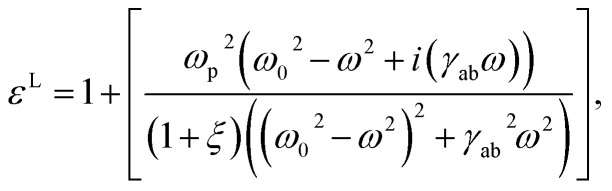
while, the nonlinear dielectric function gives,14
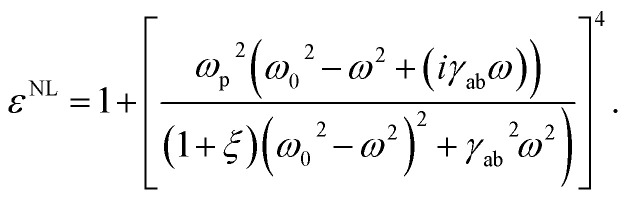


The light–matter interaction encounters either loss of light by transforming to heat, called the absorption cross-section (*δ*_abs_), or is redirected in a different direction, called the scattering cross-section (*δ*_sca_). They can be defined in terms of the dielectric function of materials using the Clausius–Mossotti relation as,^46^15
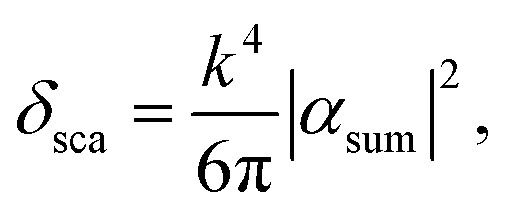
and16
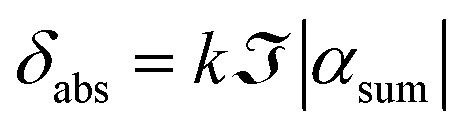
Here, *k* is the wave number, 
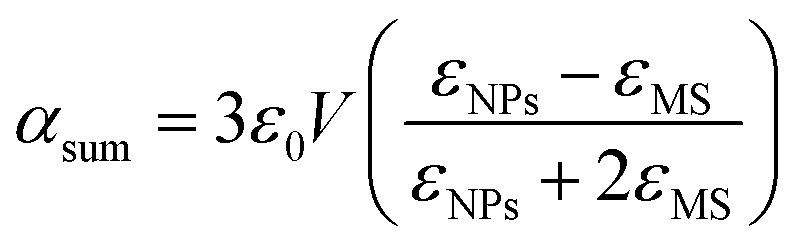
 is the polarizability of the material with *V* = 4/3π*r*^3^, the volume of spherically shaped MNPs (*r* is the radius of MNPs), *ε*_NPs_ is the linear (eqn (13)) or nonlinear (eqn (14)) dielectric function of MNPs, *ε*_MS_ is the dielectric function of the medium or metallic surface and 
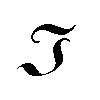
 stands for an imaginary term. With this, the total radiant flux area (extinction cross-section) is defined by a superposition of absorption and scattering cross-section as^47^

17 *δ*_exc_ = *δ*_sca_ + *δ*_abs_.

The sensitivity of the optical sensor depends on the efficiency of the SPP. That is defined as the ratio of scattering to extinction cross-section, and absorption cross-section to extinction cross-section, mathematically defined by,^48,49^18
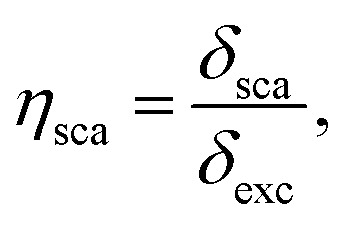
19
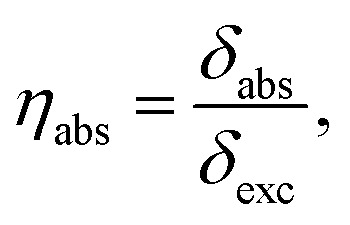
and

20 *η*_ext_ = *η*_sca_ + *η*_abs_,

where, *η*_sca_, *η*_abs_ and *η*_ext_ are scattering efficiency, absorption efficiency and extinction efficiency, respectively.

### 2.2 Quantum model of light–matter interactions

**Fig. 1:**
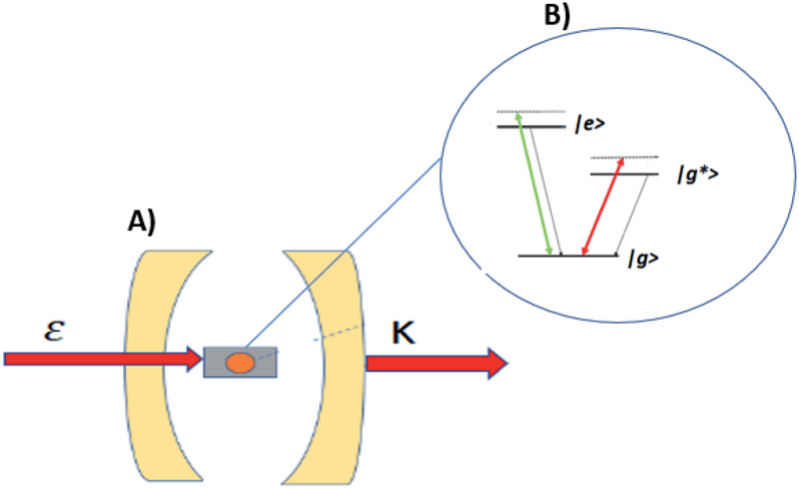
Schematic diagram of the interaction of coherent light and MNPs on MS inside a closed cavity. (A) Shows MNPs arranged on a MS inside a closed cavity. (B) Shows the V-type energy level of an atom resulting from pumping the MNPs with intense coherent light.

Enhancing light–matter interactions is required to enhance optoplasmonic biosensors. This section deals with the quantum mechanical model of emitted SPP (see Fig. 1) to study its quantum properties by employing the Hamiltonian of the system and quantization of both light and matter, from which all dynamical equations of a system are defined.

Fig. 1 illustrates a model for three level light–matter interactions denoted by |e〉, |g〉 and |g*〉 that represent the excited state, first ground state and second ground state, respectively, with possible transitions between |e〉 ↔ |g〉 and |e〉 ↔ |g*〉, but forbidden for |g〉 ↔ |g*〉.

The Hamiltonian of a system through the quantization of light and matter was stated by Jaynes and Cummings as^50^

21 *Ĥ* = *Ĥ*_F_ + *Ĥ*_M_ + *Ĥ*_Int_.

Here, *Ĥ*_F_, *Ĥ*_M_, *Ĥ*_Int_ are the field, matter and light–matter interactions Hamiltonian, and using Appendix B we can set the Hamiltonian of the system as22
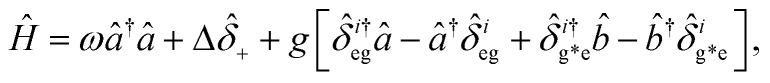
where 
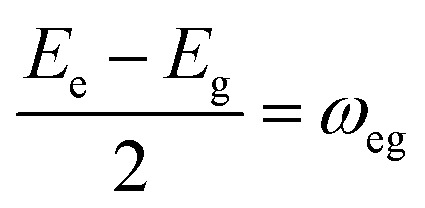
giving 
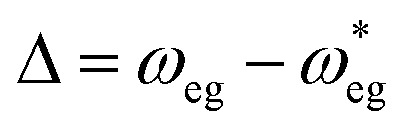
 detuning; and 
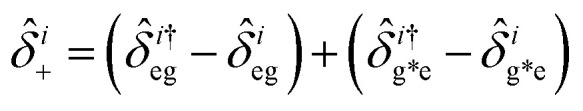
, with atomic ladder operators 
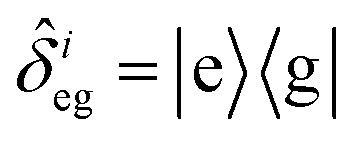
, 
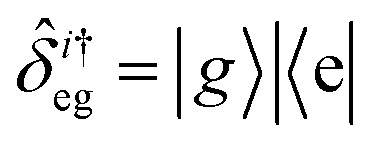
, 
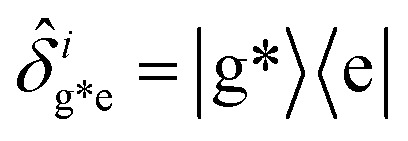
 and 
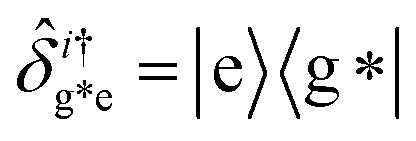
. Then, 
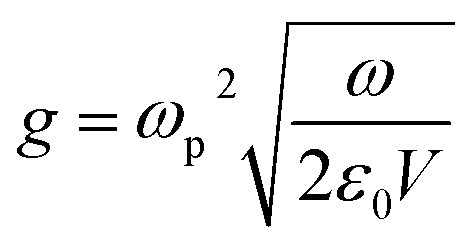
 gives the coupling between the field and MNPs, with *V* the quantized volume of MNPs that results in
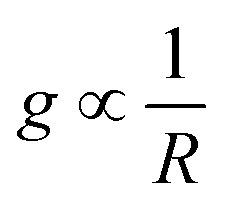
 where *R* is the radius of MNPs, implying that the smaller the radius the stronger the coupling with strong quantum properties. The plasma frequency 
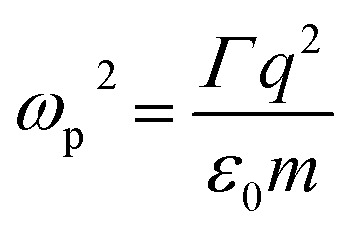
 in which *Γ* = *r̂*_12_ + *r̂*_32_.

### 2.3 Dynamical equations of a system

The dynamical equations are given by the Heisenberg–Langevin equations of evolution of atomic operators applying a quantized system as reported in ref. 51, 
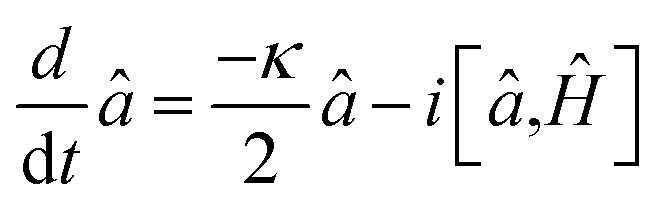
, where *κ* is the cavity damping constant and applying commutation relations, [*â*,*â*] = [*â*,*b̂*] = [*â*,*δ̂*_+_] = 0, with [*â*,*â*^†^] = [*b̂*,*b̂*^†^] = 1 the quantum Langevin equations for evolution of the atomic operator^52^ take the form (for details see Appendix C),23
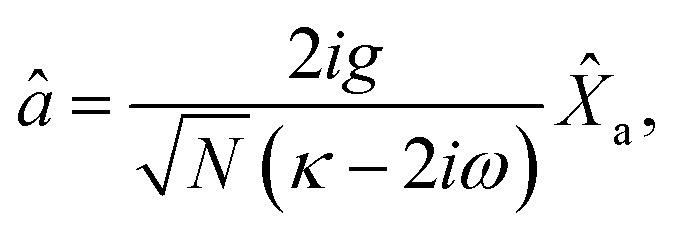
24
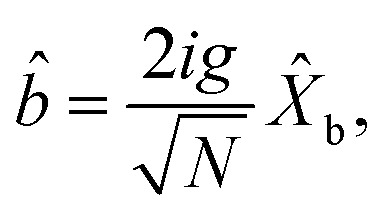
where, 
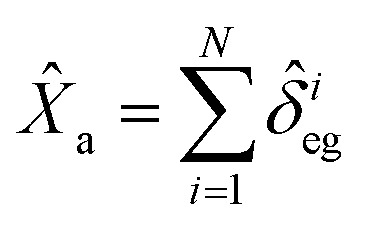
 and 
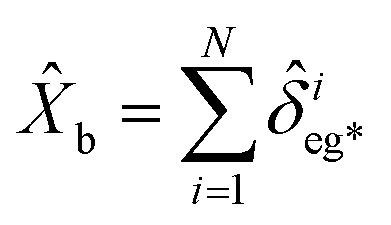
, and25
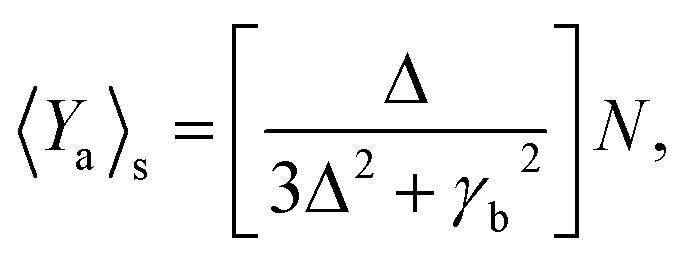
26
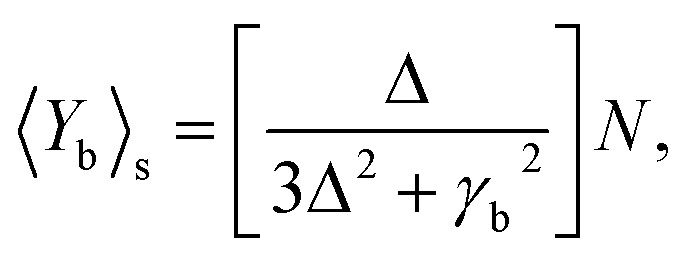
and27
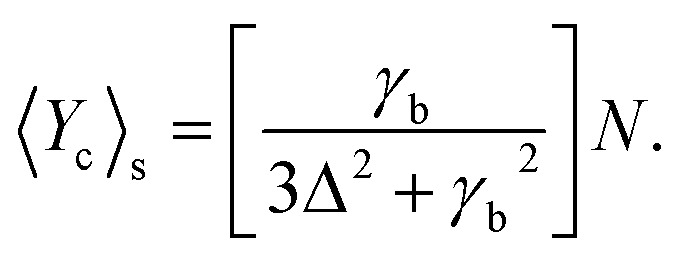
For 
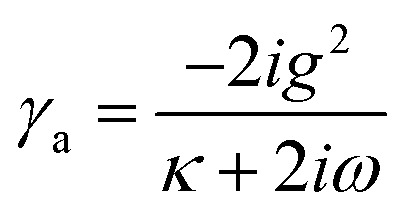
, 
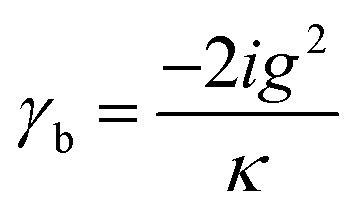
, 
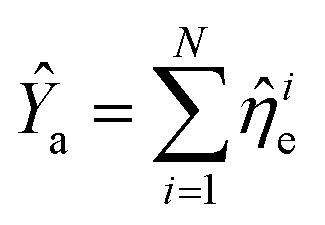
, 
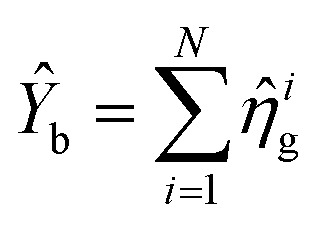
, 
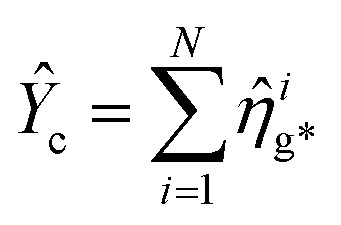
 and *N* = 〈*Ŷ*_a_〉 + 〈*Ŷ*_b_〉 + 〈*Ŷ*_c_〉.

### 2.4 Quantum properties of MNP based SPPs

SPPs that have a quantum nature have enhanced performance for microscopy, target detection, and phase estimation.^53^ The basic quantum features are defined by degrees of squeezing, quantum correlation, and preservation of entanglement^54^ as stated in the following subsections.

#### 2.4.1 Squeezing properties

The squeezing properties of light are described by quadrature squeezing and quadrature variance given by^55^

28 (Δ*ĉ*_±_)^2^ = 〈*ĉ*_+_*ĉ*_−_〉^2^.

where

29 *ĉ*_+_ = *ĉ*^†^ + *ĉ*,

30 *ĉ*_−_ = *i*(*ĉ*^†^ − *ĉ*),

Here, *ĉ* = *â* + *b̂*. Then using eqn (29) and (30), eqn (28) can be rewritten as,

31 (Δ*ĉ*_±_)^2^ = 2(1 + 〈*â*^†^*â*〉 + 〈*b̂*^†^*b̂*〉 + 〈*â*^†^*b̂*〉 + 〈*b̂*^†^*â*〉) ± 2(〈*âb̂*〉 + 〈*â*^†^*b̂*^†^〉).

On account of eqn (23) and (24) we also have,

32 (Δ*ĉ*_±_)^2^ = 2[1 + *r*_a_Δ^2^(〈*Y*_a_〉 − 〈*Y*_b_〉)^2^ − *r*_b_Δ^2^(〈*Y*_a_〉−〈*Y*_c_〉)^2^ +Δ^2^〈*Y*_a_〉(〈*Y*_a_〉 − 〈*Y*_b_〉 − 〈*Y*_c_〉) + Δ^2^〈*Y*_c_*Y*_b_〉] ±4[Δ^2^〈*Y*_a_〉(〈*Y*_a_〉 − 〈*Y*_b_〉 − 〈*Y*_c_〉) + Δ^2^〈*Y*_c_*Y*_b_〉].

Then, incorporating eqn (25)–(27) into eqn (31), plus and minus quadrature take the form33
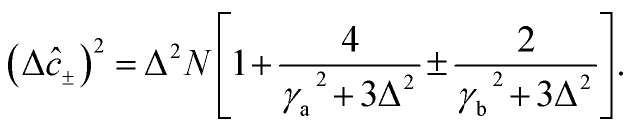


From quadrature variance, we can derive quadrature squeezing as34
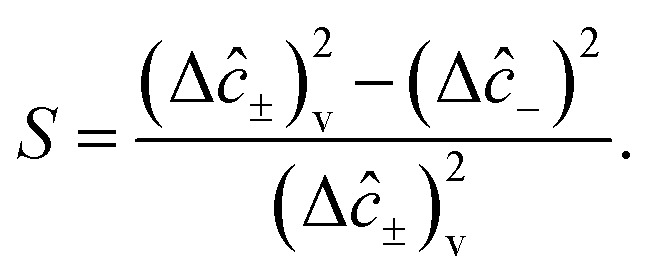


#### 2.4.2 Entanglement properties

The first inseparability condition proposed by Simon and Duan is the sufficient conditions for quantification of entanglement in a two-mode CV system. The criterion suggest that if the two modes are separable, they should satisfy the inequality:^56^

35 Δ*û*^2^ + Δ*v̂*^2^ ≤ 2*N*.

For, *û* = *m*_1_ − *m*_2_, and *v̂* = *m*′ − m*; with 
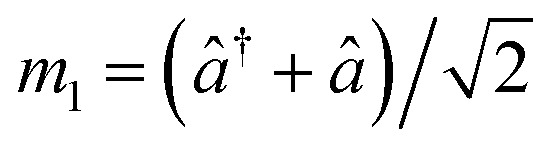
, 
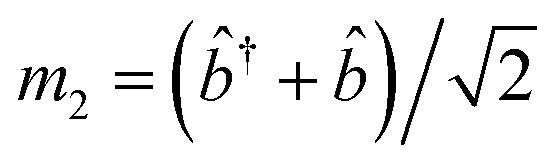
, 
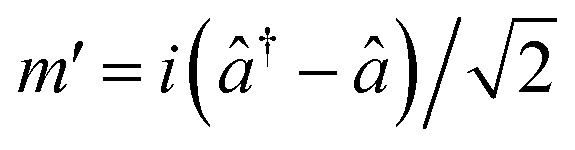
 and 
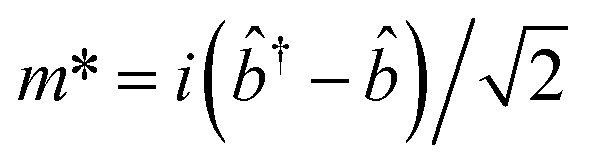
.

Substituting eqn (23) and (24) into eqn (35) the Duan–Giedke–Cirac–Zoller (DGCZ) criterion takes the form,36
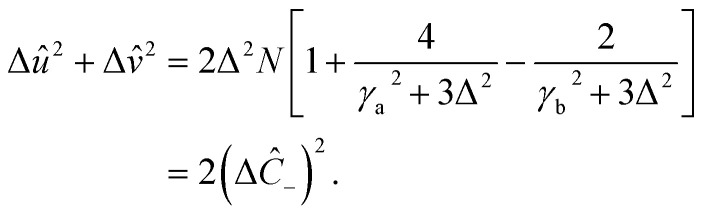


#### 2.4.3 Second order correlation function

The second order correlation function is the major feature to distinguish non-classical, anti-bunching light sources from classical light.^57^ It plays an instrumental role in the construction of advanced biosensors since it is used for quantum information, cold atomic cloud, spectroscopy of quantum dots and fluorescence correlation.^58^ Mathematically, the second order correlation function is defined by^59^37
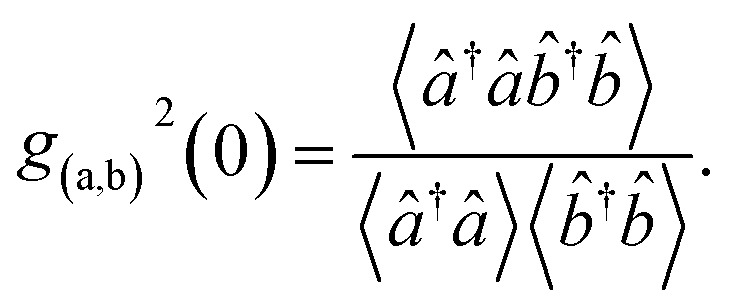


Since the expectation of both beams of light are Gaussian variable with zero means, eqn (37) takes the form38
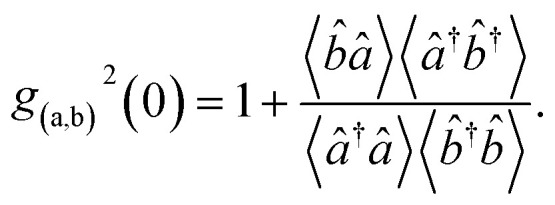


On account of eqn (23) and (24) the second order correlation function is given by,39
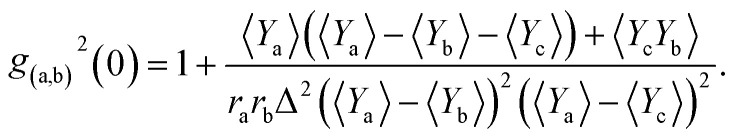


Then, incorporating eqn (25)–(27) into eqn (39) the second order correlation function takes the form40
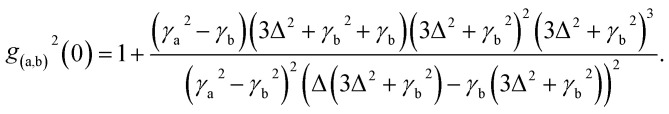


## 3 Results and discussion

**Fig. 2:**
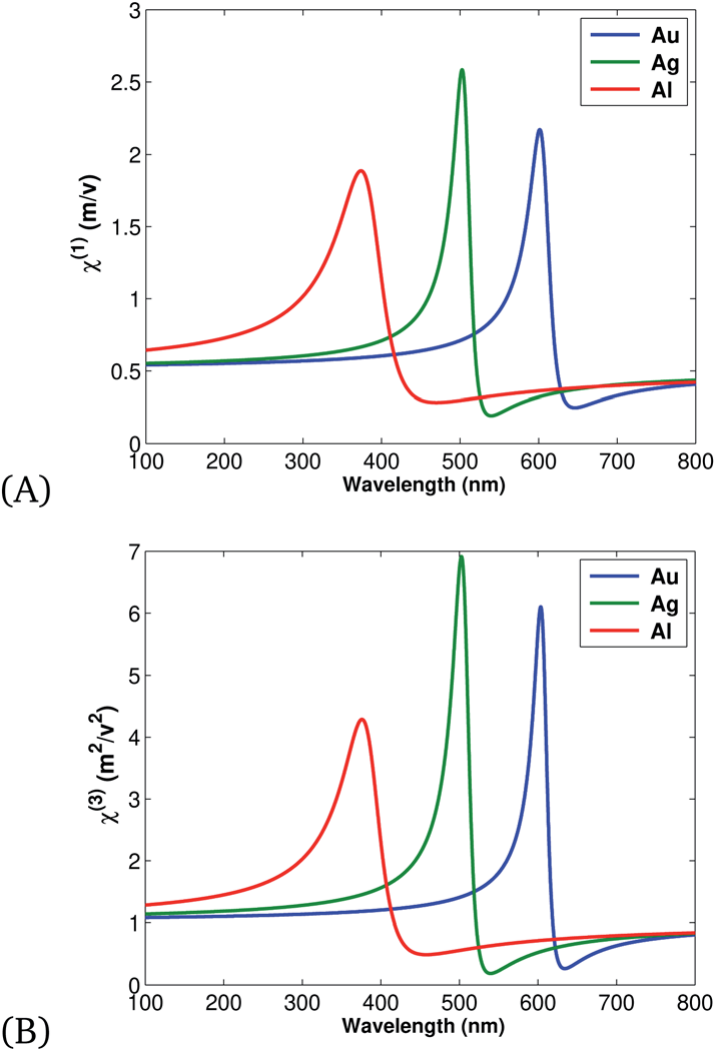
Variation of (A) linear (eqn (9)) and (B) nonlinear (eqn (10)) intensity for Au, Ag, and Al, plotted using the parameters presented in Table 1.

The classical and quantum model of the SPP was studied using Maxwell equations, the Drude model, and by quantizing the resulting field from laser light and Ag/Fe_2_O_3_, Au/Fe_2_O_3_, Al/Fe_2_O_3_ interactions. The classical model result revealed that the nonlinear optical properties of materials have more intensity than the linear (as illustrated in Fig. 2), since more variation of the induced electronic polarization occurs. Additionally, out of all nobel MNPs, Au has more intensity due to more free electrons and so the result is in agreement with the experimental work of ref. 64.

**Fig. 3:**
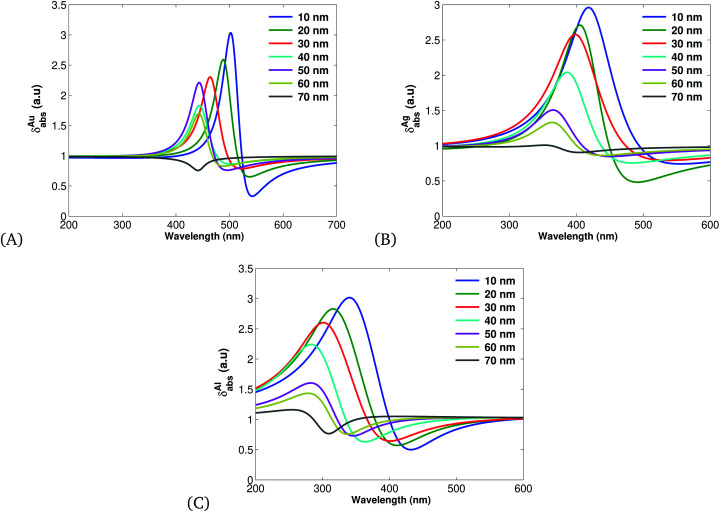
Plots of absorption cross sections (eqn (16)) plotted using the parameters presented in Table 1 for different values of *r.* (A) for Ag, (B) for Au and (C) for Al.

Further, employing the nonlinear susceptibility of materials (eqn (14)) we have investigated the absorption cross section (see Fig. 3A–C) of nobel MNPs. It is found that increasing the size of MNPs decreases the absorption cross-section spectra and the aluminium based SPPs have more absorption cross section in comparison with other nobel MNPs, thus comply with the experimental and theoretical work of ref. 65 and 66.

**Fig. 4:**
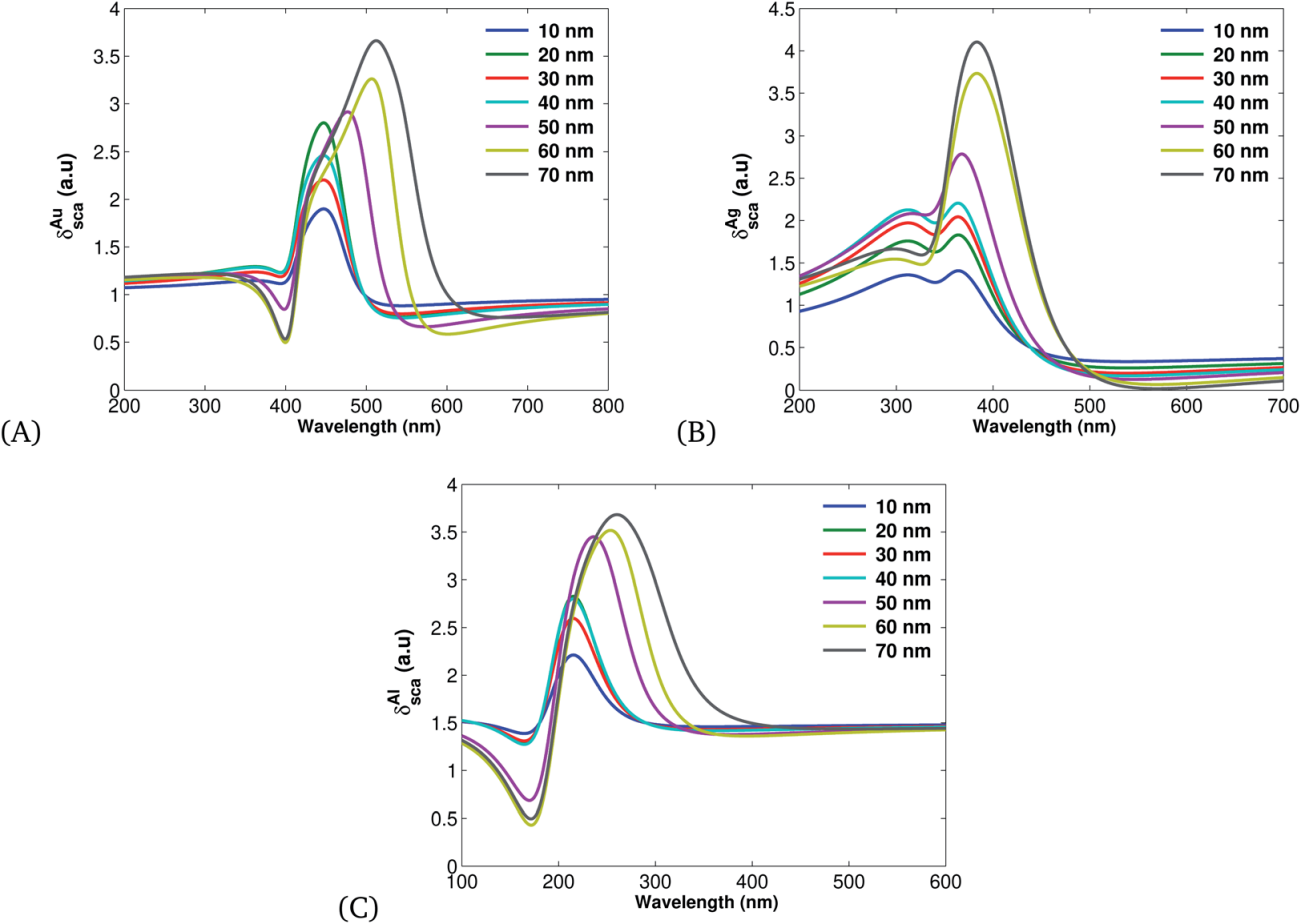
Plots of scattering cross sections (eqn (15)) plotted using the parameters listed in Table 1 for different values of *r* and *λ* = 632.8 nm: (A) for Au, (B) Ag and (C) for Al.

The additional measurement of rate at which a particular light–matter interaction occurs has a scattered cross section defined by eqn (15). Fig. 4 displays the scattering cross section of Au (Fig. 4A), Ag (Fig. 4B) and Al (Fig. 4C) under different spherical MNP sizes. Fig. 4 clearly shows that the scattering cross section increases with the size of MNPs, and Ag has a larger scattering cross section than Au or Al (Table 1).

**Table 1:** Optical parameters – plasma frequency (*ω*_p_), dielectric function (*ε*) and damping constant (*γ*_ab_) – for Fe_2_O_3_, Al, Au, Ag

Material	*ω*_p_	*ε*	*γ*_ab_	Ref.
Fe_2_O_3_	21.451	12.457	0.749	60
Al	15.416	9.481	0.796	61
Au	13.973	7.264	0.646	62
Ag	13.716	5.763	0.453	63

**Fig. 5:**
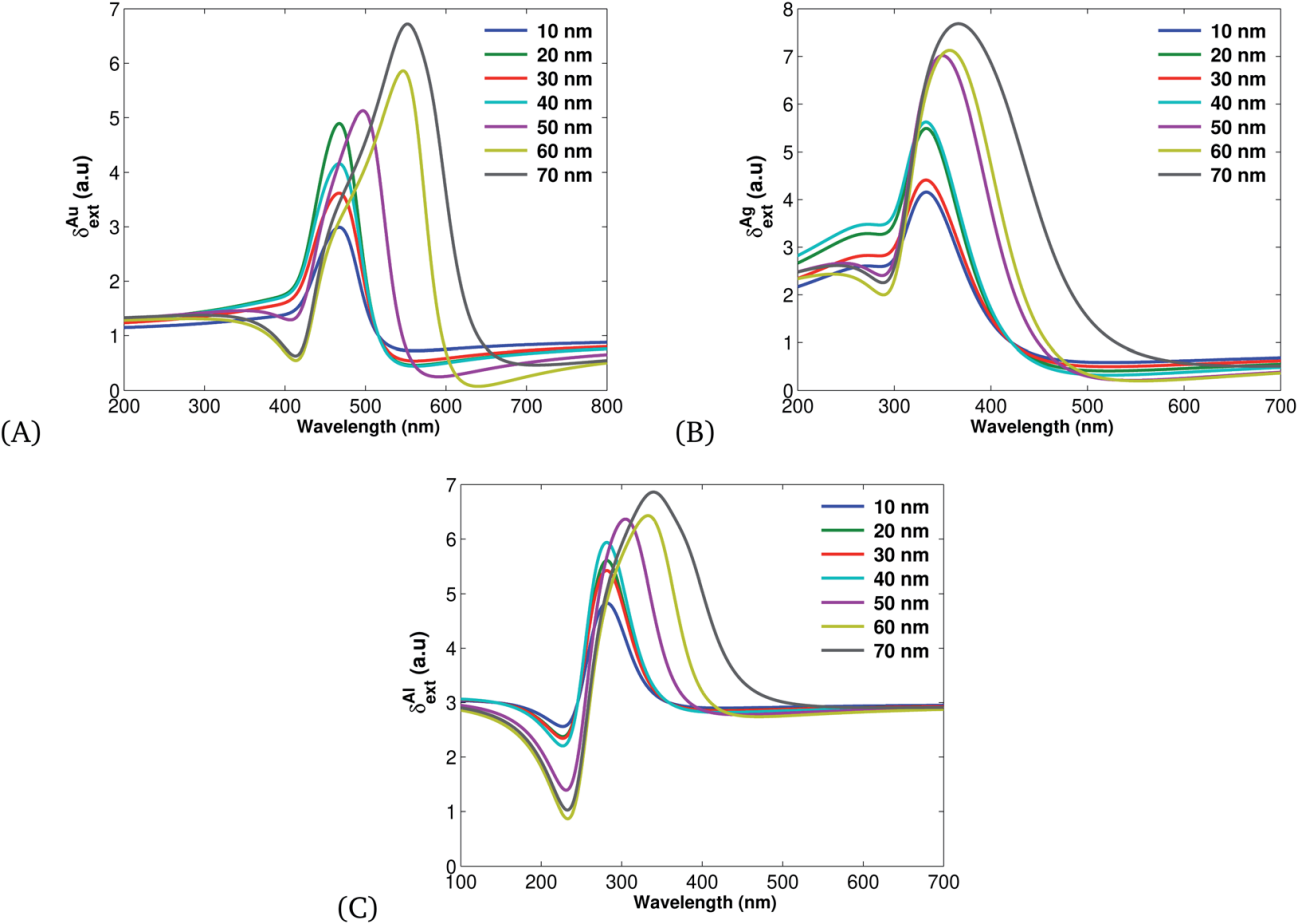
Plots of extinction cross sections (eqn (17)) plotted using the parameters listed in Table 1 for different values of *r*. (A) for Au, (B) Ag and (C) for Al.

Fig. 5 shows the extinction cross section and it exceeds both the absorption and scattering cross sections since it is the sum of the two. This work is in agreement with other theoretical reports.^67^

Therefore, on account of eqn (18)–(20) and Fig. 3–5 we can summarize that Ag-based SPPs have more overall efficiency than Au and Al.

### 3.1 Quantum feature measurement

In quantum mechanical principles, the full picture of the physical observable cannot be captured in a single measurement, rather the detection has to be performed many times under the same preparation conditions and taking the expected value. Squeezed, correlated and entangled sources of light are used to reduce noise by phase matching techniques to measure elasticity, ionization, and bond type (covalent, ionic, and hydrogen bonds) of a cell. From the classical model we illustrate that Ag-based SPPs have higher efficiency, therefore, in the next subsections we focus on the quantum features of Ag-based SPPs.

**Fig. 6:**
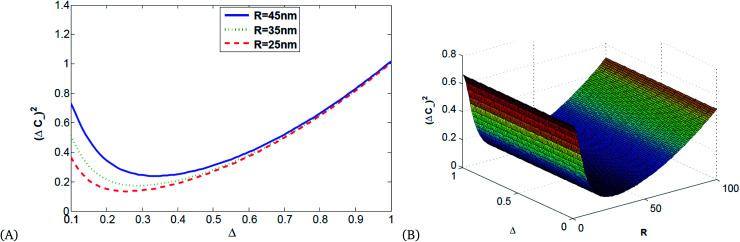
(A) 2D and (B) 3D illustrations of quadrature variance *vs.* detuning and the radius of Ag MNPs for *κ* = 0.8, using the parameters presented in Table 1.

Fig. 6 shows that squeezing occurs in the minus quadrature, and quadrature variance depends on size and detuning. Quadrature variance increases with detuning and frequency but decreases as the size of MNPs increases, this indicates smaller particles (10–40 nm) have quantum features rather than bulk materials.

**Fig. 7:**
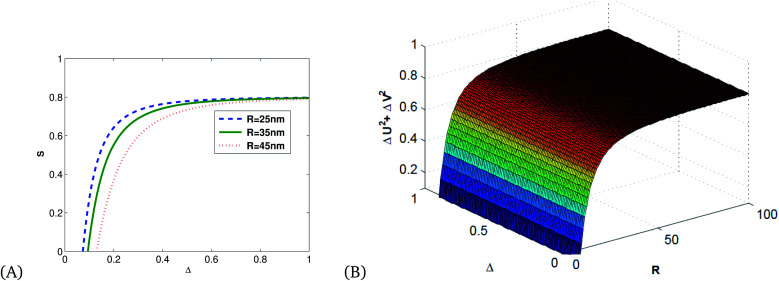
(A) 2D and (B) 3D illustrations of quadrature squeezing *vs.* detuning and the radius of Ag NPs for *κ* = 0.8, *ω*_p_ = 13.716.

Fig. 7 shows the direct proportionality of the degree of squeezing with detuning, size of the Ag NPs, and frequency.

**Fig. 8:**
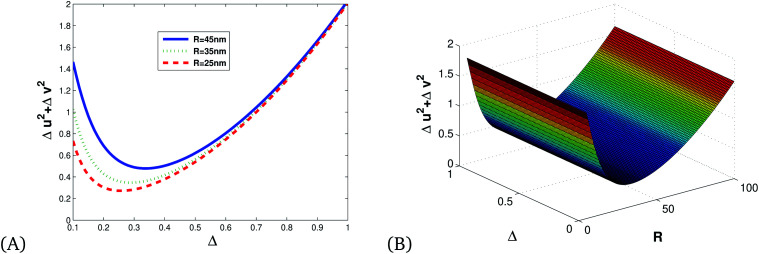
(A) 2D and (B) 3D illustrations of entanglement *vs.* detuning and the radius of single MNPs for *κ* = 0.8, *ω*_p_ = 13.716 and *λ* = 632 nm.

Fig. 8 is an illustration of entanglement in 2D and 3D that shows that the degree of entanglement is proportional to detuning but decreases as the size of MNPs increases. This shows that detuning is proportional to relaxation time, and as the size of MNPs decreases relaxation time increases since the polariton (collective free electron) can propagate through tiny particles freely.

**Fig. 9:**
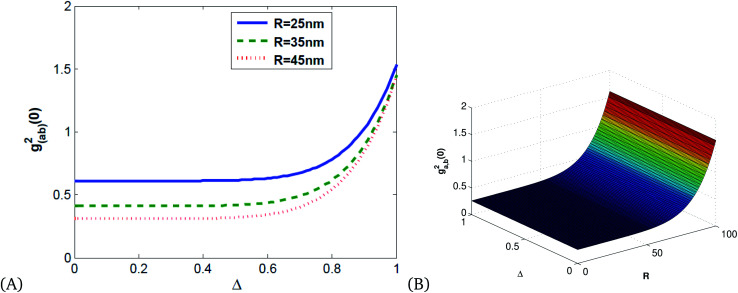
Plots of quadrature squeezing *vs.* detuning and frequency for different radii of single Ag NPs for *κ* = 0.8, *ω*_p_ = 13.716.

Fig. 9 shows that the degree of correlation increases with detuning and decreases as the size of Ag NPs increases, since the size of material increases, the absorption of light leads to the production of thermal energy that results in noise.

**Fig. 10:**
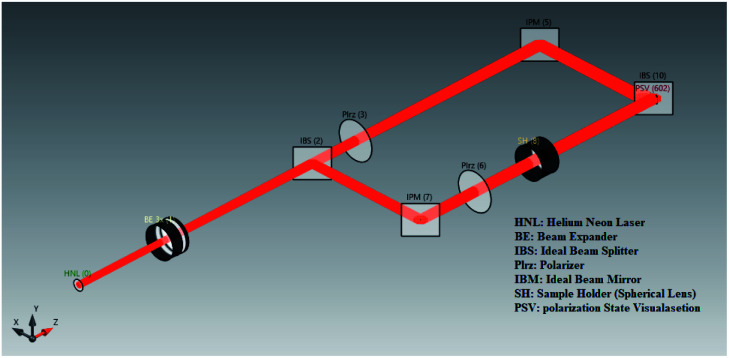
Visual of the components of OPBs using the raytracing system analyzer (RSA) simulation engine of VirtualLab Fusion software.

Therefore the Rabi splitting with active control expressed by Wen *et al.*^68^ has a significant impact for controlling temperature, but it is not enough to cool and control the biosensor for study both experimentally and theoretically. We introduce nonlinear-based OPS with five main components as shown in Fig. 10, which generates bright light. The components are arranged as follows: light source, different types of lenses for controlling light, object (sample holder), detector (interaction of organisms/light with different molecular species) and visual screen (gives the output of measurements).

The efficiency of the SPP field is described by the propagation length (*L*_*x*_) and penetration depth (*L*_*y*_) as light propagates along the *z*-axis,^69–71^ expressed mathematically41
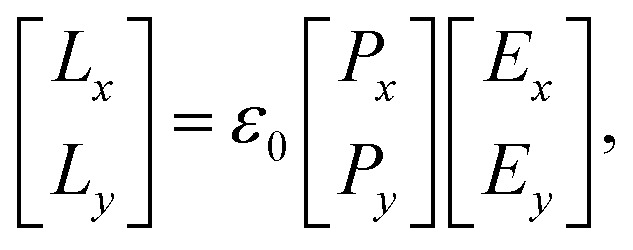
where, *E*_*x*_ and *E*_*y*_ are the incident field along the *x* and *y*-axis with42
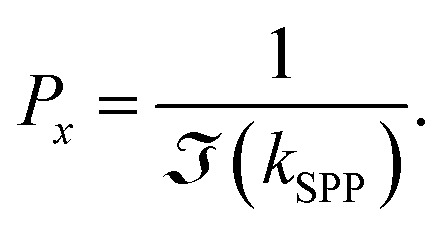
and43
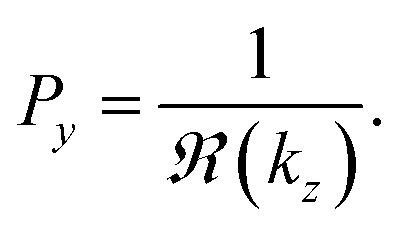
Here, 
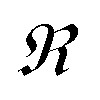
 and 
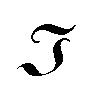
 are the real and imaginary parts of the dielectric function for44
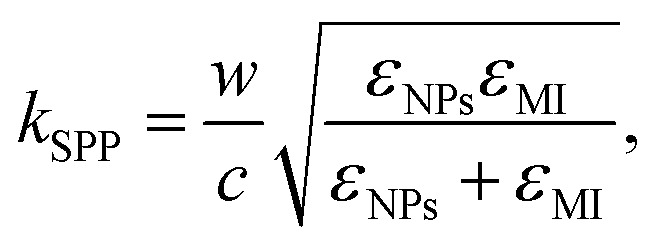
and45
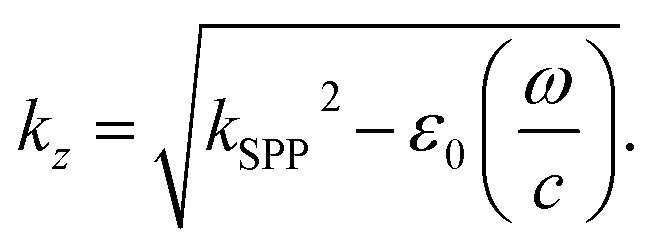


**Fig. 11:**
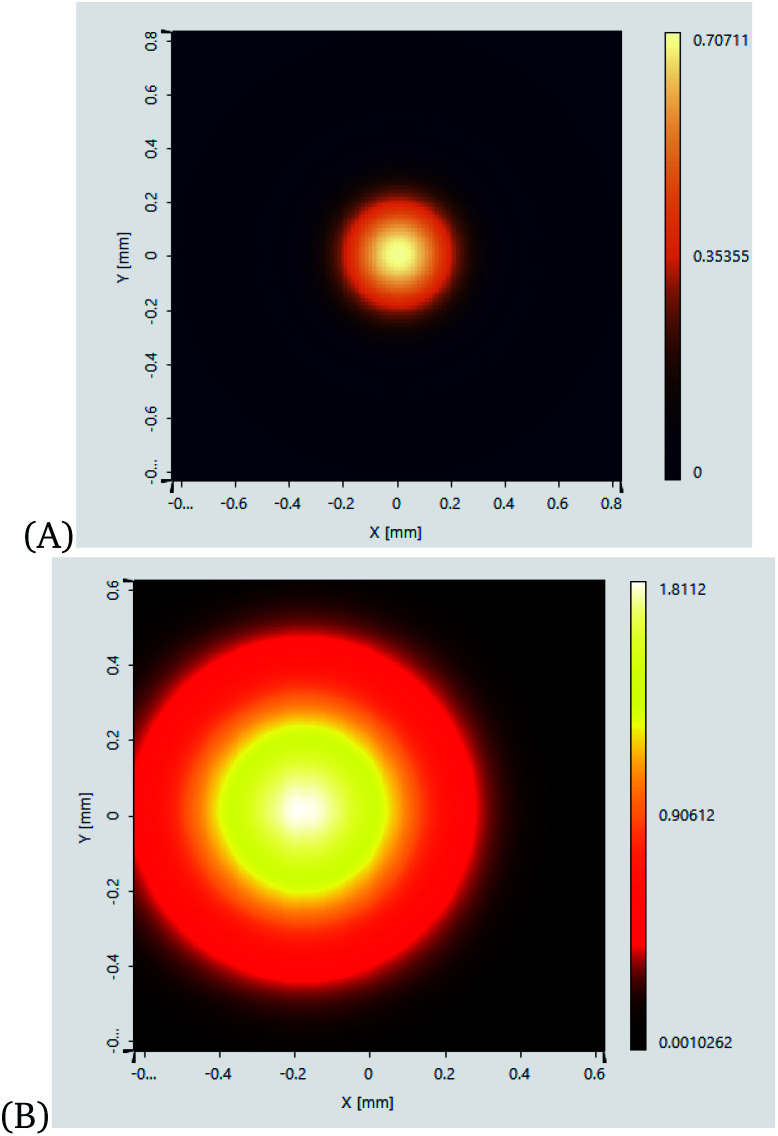
Output measurement of light with and without SPP in a vacuum reservoir using model analyzer (RSA) commercially available simulation engine of VirtualLab Fusion software^72,73^ applying eqn (41): (A) without SPP, (B) amplified by SPP.

The quantum features of light are strong and sensitive enough to measure cell mechanics. Cell mechanics is the generalized name for the differentiation of a cell, morphological alterations of the cell, and cell cycle. Measurement of cell mechanics under sensitive instruments like SPP-based OPBs helps to distinguish cancer cells from normal ones. The output measurements of light with and without SPPs are presented in Fig. 11.

Fig. 11 shows the enhancement of the incident field with MNPs (Ag) enhancing the sensitivity and selectivity of OPS. We note from this figure that with the brightness of incident light, if any biological sample, especially cancer cells, are added the obtained brightness is enough to show the cell mechanics of cancer cells.

## 4 Conclusions

In this work, we have established the Nobel classical and quantum approach to model optical properties of MNPs for the sensitivity enhancement of optical biosensors. With the help of the Maxwell equations and modified Drude model, we have obtained equations of the dielectric function of materials from which relaxation time, absorption efficiency, and extinction efficiency, comparing linear with nonlinear optical properties, of SPPs are derived. Following this, we have obtained the Hamiltonian of a system by quantizing the equation of propagation that leads to obtaining the quantum Langevin equation to test the quantum nature of the resulting SPP from light–matter interactions. From this work, it can be noted that the sensitivity of the optoplasmonic biosensor increases as the size of stimulants (noble MNPs) decreases, Ag NPs are more efficient for sensitivity and selectivity enhancement in OPS compared with other MNPs. In addition, we have also found that nonlinear SPPs have a high-efficiency quantum nature that contributes to controlling noise in OPS. Finally we conclude that the nonlinear optical properties of a Ag-based surface plasmon polariton results in bright light with sensitive, selective, and low cost OPS that could be used to image and treat cancer cells.

## Appendix

### A Output field

A.1 *E*_out_(*ω*,*t*) = *E*_in_(*ω*,*t*) − (*E*_abs_(*ω*,*t*) + *E*_tra_(*ω*,*t*))

where,A.2
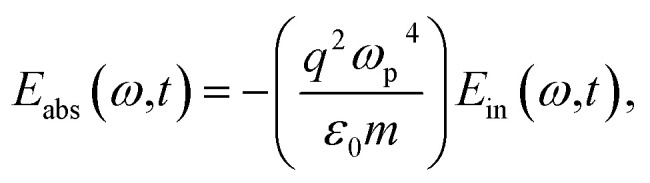
A.3
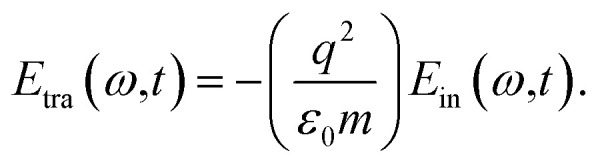


### B Derivation of Hamiltonian


B.1 *Ĥ* = *Ĥ*_F_ + *Ĥ*_M_ + *Ĥ*_Int_,

B.2 *E*_in_(*r*,*t*) → *Ê*_in_(*r*,*t*),

B.3

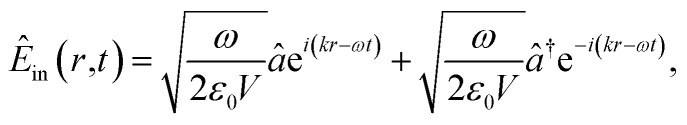




B.4

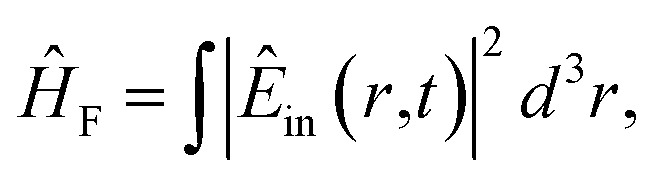




B.5 *Ĥ*_F_ = *ωââ*^†^.

B.6

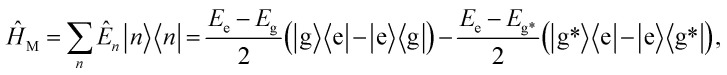




B.7 *Ĥ*_M_ = Δ*δ̂*^*i*^_+_,

B.8

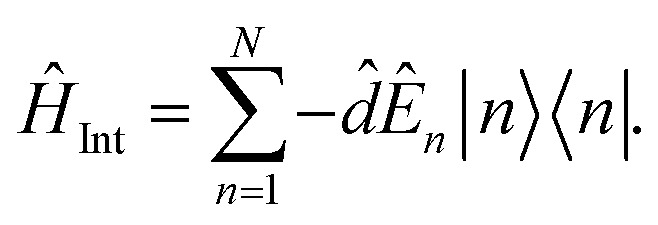



In which, the dipole excited by the incident electric field is

B.9 *d̂* = *α*|*Ê*_in_(*r*,*t*)|^2^,

B.10
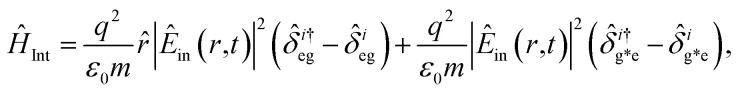
B.11
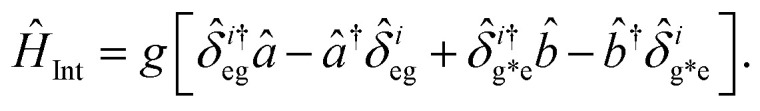


### C Derivation of equations of evolution of operators

Employing the quantum Langevin equation by substituting the top and bottom level atoms we findC.1
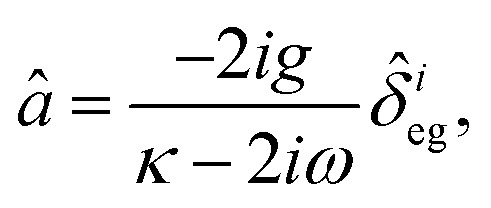
andC.2
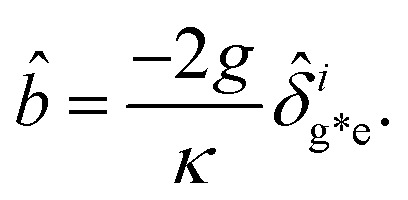


Considering the commutation relation of atomic operators, 
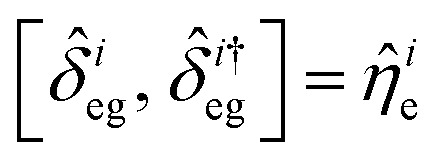
, 
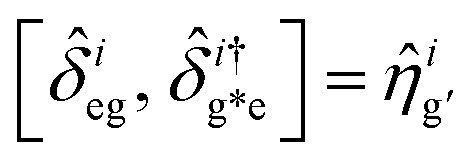
 and 
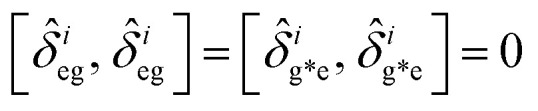
 we can set equations of atomic operators asC.3
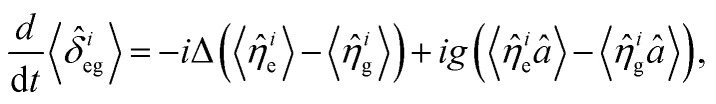
C.4
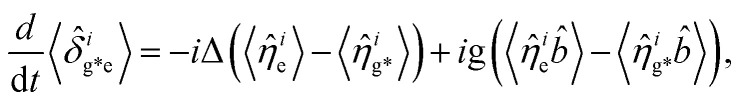
C.5
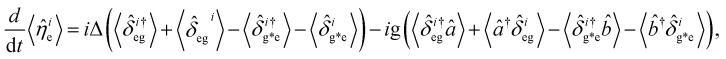
C.6
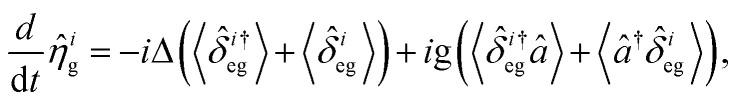
andC.7
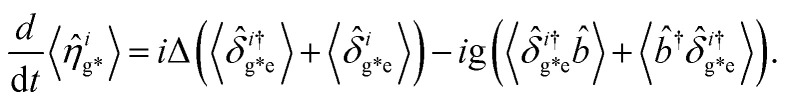
C.8
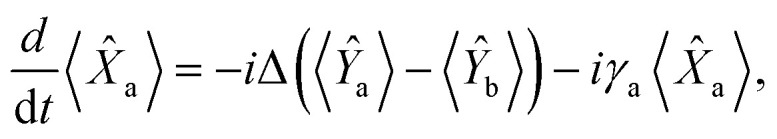
C.9
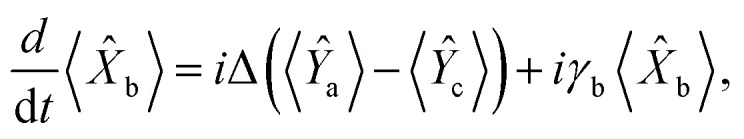
C.10
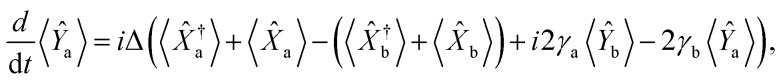
C.11
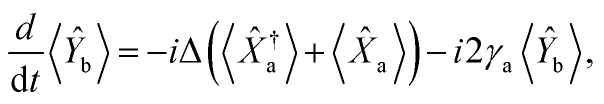
C.12
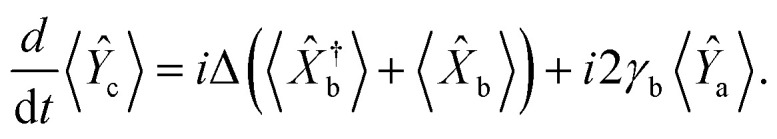


From this we can setC.13
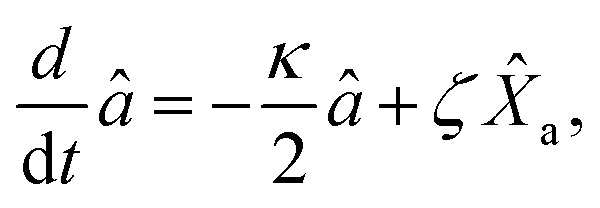
andC.14
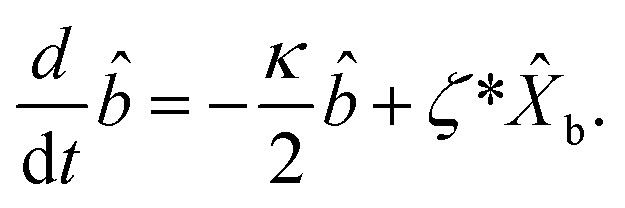


Taking a large time approximation we find,C.15
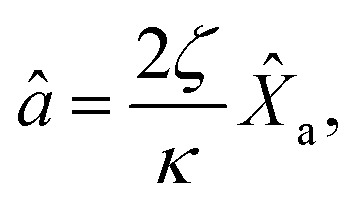
andC.16
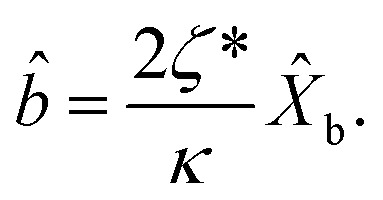


Using the commutation relation of eqn (C.15) and (C.16) with its conjugate we have,C.17
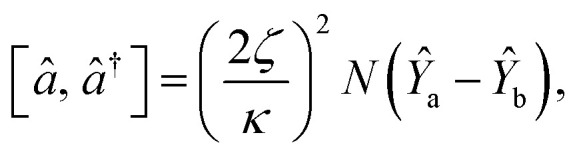
andC.18
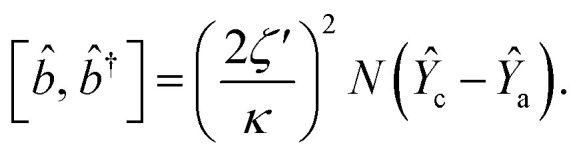


Taking the commutation relation of eqn (C.1) and (C.2) with its complex conjugate gives,C.19
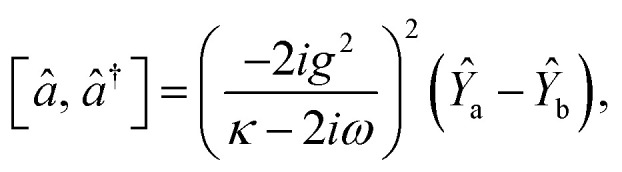
andC.20
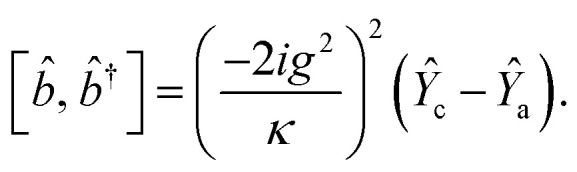


Equating the above commutation relations gives,C.21
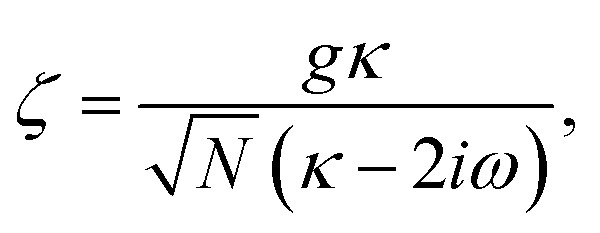
andC.22
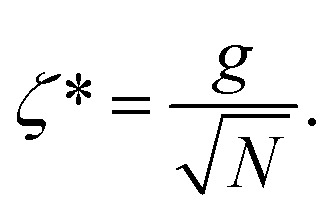


## Author contributions

AG: carried out theoretical modelling; AB: developed computational modelling and edited the manuscript; TA: carried out theoretical modelling and AK: edited the manuscript;

## Conflicts of interest

There are no conflicts to declare.

## Supplementary Material

## References

[cit1] Rivera N., Kaminer I. (2020). Nat. Rev. Phys..

[cit2] Davies P. (2020). Phys. Today.

[cit3] England J. L. (2013). J. Chem. Phys..

[cit4] Lam K. S. , Fundamental Principles of Classical Mechanics. A Geometrical Perspective, California State Polytechnic University, Pomona, USA, 2014, vol. 121923, pp. 1–8

[cit5] Xu L., Rahmani M., Ma Y., Smirnova D. A., Kamali K. Z., Deng F., Chiang Y. K., Huang L., Zhang H., Gould S., Neshev D. N., Miroshnichenkoa A. E. (2020). Adv. Photonics.

[cit6] Li T. E., Li T. E. (2019). J. Chem. Theory Comput..

[cit7] Chen M., Kurman Y., Karnieli A., Rivera N., Arie A., Kaminer I. (2021). Optica.

[cit8] Rahman A. U., Noman M., Javed M., Ullah A., Luo M. (2021). Laser Phys..

[cit9] Mohamed A., Hessianb A., Cimini E. V., Mellini M., Rampioni G., Sbroscia M. (2020). Chaos, Solitons Fractals.

[cit10] Mahdizadeh S. J., Goharshad E. h K. (2020). RSC Adv..

[cit11] Gao X., Deng Y. (2020). Int. J. Intell. Syst..

[cit12] Webb J. L., Troise L., Hansen N. W., Olsson Ch., Wojciechowski A. M., Achard J., Brinza O., Staacke R., Kieschnick M., Meijer J., Thielscher A., Perrier J., Sørensen K. B., Huck A., Andersen U. L. (2021). Sci. Rep..

[cit13] Wen G., Zou X., Hu R., Peng J., Chen Z., He X., Dong G., Zhang W. (2021). RSC Adv..

[cit14] Cong S., Liu X., Jiang Y., Zhang W., Zhao Z. (2020). Innovation.

[cit15] Hu G., Shen J., Qiu C. W., Alù A., Dai S. (2020). Adv. Opt. Mater..

[cit16] Dombi P., Pápa Z., Vogelsang J., Yalunin S. V., Sivis M., Herink G., Schäfer S., Groß P., Ropers C., Lienau C. (2020). Rev. Mod. Phys..

[cit17] El-Sharkawy Y. H., Elbasuney S., Radwan S. M., Askar M. A., El-Sayyad G. S. (2021). RSC Adv..

[cit18] Wang L., Wang Z., Li L., Zhang J., Liu J., Hu J., Wu X., Weng Z., Chu X., Li J., Qiao Z. (2020). RSC Adv..

[cit19] Gubbin C. R., De Liberato S., Folland T. G. (2022). J. Appl. Phys..

[cit20] Mpofu K. T., Lee C., Maguire G. E., Kruger H. G., Tame M. S. (2022). J. Appl. Phys..

[cit21] Galiffi E., Tirole R., Yin S., Li H., Vezzoli S., Huidobro P. A., Silveirinha M. G., Sapienza R., Alù A., Pendry J. B. (2022). Adv. Photonics.

[cit22] Lu X., McClung A., Srinivasan K. (2022). Nat. Photonics.

[cit23] Hamid A., Zafar A., Liaqat I., Afzal M. S., Peng L., Rauf M. K., ul Haq I., Ali S., Aftab M. N. (2022). RSC Adv..

[cit24] Xie R. C., Tsay S. Y., Wu J. J., Kuo C. C., Zhang H., Lin J. H. (2022). Opt Laser. Technol..

[cit25] Luo X., Tsai D., Gu M., Hong M. (2019). Chem. Soc. Rev..

[cit26] Chen X., Lindley-Hatcher H., Stantchev R. I., Wang J., Li K., Hernandez Serrano A., Taylor Z. D., Castro-Camus E., Pickwell-MacPherson E. (2022). Chem. Phys. Rev..

[cit27] Hang Y., Boryczka J., Wu N. (2022). Chem. Soc. Rev..

[cit28] Mierke C. T. , in The Physics of Cancer: Research Advances, 2021, pp. 101–134

[cit29] Haase K., Pelling A. E. (2015). J. R. Soc., Interface.

[cit30] Wala J., Das S. (2020). Biomech. Model. Mechanobiol..

[cit31] Runel G., Lopez-Ramirez N., Chlasta J., Masse I. (2021). Cells.

[cit32] Huang X., Song J., Yung B. C., Huang X., Xiong Y., Chen X. (2018). Chem. Soc. Rev..

[cit33] Zhang Z., Ding H., Yan X., Liang L., Wei D., Wang M., Yang Q. (2018). Opt. Mater. Express.

[cit34] Karadurmus L., Ozcelikay G., Vural S., Ozkan S. A. (2021). Iran. J. Pharm. Res..

[cit35] Crowell L. L., Yakisich J. S., Aufderheide B., Adams T. N. (2020). Micromachines.

[cit36] Mizrahi J. D., Surana R., Valle J. W., Shroff R. T. (2020). Lancet.

[cit37] Mauri F., Schepkens C., Lapouge G., Drogat B., Song Y., Pastushenko I., Rorive S., Blondeau J., Golstein S., Bareche Y., Miglianico M. (2021). Nat. Cancer.

[cit38] Qian L., Li Q., Baryeh K., Qiu W., Li K., Zhang J., Yu Q., Xu D., Liu W., Brand R. E., Zhang X. (2019). Transl. Res..

[cit39] Weltin A. , Kieninger J. and Urban G. A., in Multidisciplinary Digital Publishing Institute Proceedings, 2017, vol. 1, p. 521

[cit40] Bellassai N., D’Agata R., Jungbluth V., Spoto G. (2019). Front. Chem..

[cit41] Vindas K., Buhot A., Livache T., Garrigue P., Sojic N., Leroy L., Engel E. (2020). Opt. Express.

[cit42] Rivera N., Kaminer I. (2020). Nat. Rev. Phys..

[cit43] Frezza F., Mangini F., Tedeschi N. (2018). J. Opt. Soc. Am. A.

[cit44] Zeng W., Gong S., Zhong C., Yang C. (2019). J. Phys. Chem. C.

[cit45] Alabastri A., Tuccio S., Giugni A., Toma A., Liberale C., Das G., Angelis F. D., Fabrizio E. D., Zaccaria R. P. (2013). Materials.

[cit46] Frumar M. , Wagner T., Shimakawa K. and Frumarova B., in Nanomaterials and Nanoarchitectures, 2015, pp. 151–238

[cit47] Nima Z. A., Davletshin Y. R., Watanabe F., Alghazali K. M., Kumaradas J. C., Biris A. S. (2017). RSC Adv..

[cit48] Navarroab J. R. G., Werts H. V. (2013). Analyst.

[cit49] Cooling L. (2015). Clin. Microbiol. Rev..

[cit50] Bina M. (2012). Eur. Phys. J.: Spec. Top..

[cit51] Ghasemian E., Tavassoly M. K. (2021). Phys. A.

[cit52] Kantorovich L., Ness H., Stella L., Lorenz C. D. (2016). Phys. Rev. B.

[cit53] Olmi S., Gori M., Donato I., Pettini M. (2018). Sci. Rep..

[cit54] Stucky G. D. , Marder S. R. and Sohn J. E., Materials for Nonlinear Optics, 1991

[cit55] Kumar P., Vedeshwar A. G. (2018). J. Opt. Soc. Am. B.

[cit56] Penndorf R. B. (1962). J. Opt. Soc. Am..

[cit57] Kheirandish A., Javan N. S., Mohammadzadeh H. (2020). Sci. Rep..

[cit58] Civitarese O., Reboiro M., Rebón L., Tielas D. (2010). Phys. Lett. A.

[cit59] Nair J. M. P., Agarwal G. S. (2020). Appl. Phys. Lett..

[cit60] Lunt R. A., Jackson A. J., Walsh A. (2013). Chem. Phys. Lett..

[cit61] Ordal M. A., Long L. L., Bell R. J., Bell S. E., Bell R. R., Alexander R. W., Ward C. A. (1983). Appl. Opt..

[cit62] Yang H. U., D’Archangel J., Sundheimer M. L., Tucker E., Glenn D. (2015). Phys. Rev. B: Condens. Matter Mater. Phys..

[cit63] Derkachova A., Kolwas K., Demchenko I. (2016). Plasmonics.

[cit64] Mo S., Guo K., Liu G., He X., Lan J., Zhou Z. (2020). Thin Solid Films.

[cit65] Alrahili M., Savchuk V., McNear K., Pinchuk A. (2020). Sci. Rep..

[cit66] Lewis T. R., Gómez Martín J. C., Blitz M. A., Cuevas C. A., Plane J., Saiz-Lopez A. (2020). Atmos. Chem. Phys..

[cit67] Shafiqaa A. R., Azizb A. A., Mehrdelc B. (2018). J. Phys.: Conf. Ser..

[cit68] Wen J., Wang H., Wang W., Deng Z., Zhuang C., Zhang Y., Liu F., She J., Chen J., Chen H., Deng S., Xu N. (2017). Nano Lett..

[cit69] Nayak J. K., Jha R. (2019). Mater. Res. Express.

[cit70] Barnes W. L. (2006). J. Opt. A: Pure Appl. Opt..

[cit71] Law S., Podolskiy V., Wasserman D. (2013). Nanophotonics.

[cit72] Ribes-Pleguezuelo P. , Zhang S., Beckert E., Eberhardt R., Wyrowski F., and Tünnermann A., Optica Publishing Group, 2017 10.1364/OE.25.005927 28381063

[cit73] Zhang S. , Proc. SPIE OP20EX, SPIE Exhibition Product Demonstrations, 2020, p. OP20EX0G

